# Prevalence and associated factors of teenage childbearing among Ethiopian women using semi-parametric and parametric proportional hazard and accelerated failure time models

**DOI:** 10.1186/s12905-024-03190-0

**Published:** 2024-06-15

**Authors:** Ashefet Agete, Mesfin M. Ayalew, Sebsebe Admassu, Zelalem G Dessie

**Affiliations:** 1 College of Science, Bahir Dar University, Bahir Dar, Ethiopia; 2 School of Mathematics, Statistics and Computer Science, University of KwaZulu-Natal, Durban, South Africa

**Keywords:** AFT model, Cox PH model, Parametric PH model, Teenage childbearing

## Abstract

**Background:**

Teenage childbearing is a common issue for young people’s sexual and reproductive health in the world, particularly in low-income countries, and affects teenagers between the ages of 13 and 19. According to several academics, adolescent pregnancy accounts for the majority of Ethiopia’s population increase, and there has been little effort to address this threat. This study aimed to determine the prevalence and associated factors of the time to teenage childbearing in Ethiopia.

**Method:**

This paper compares the results of the semi-parametric proportional hazard (PH), parametric PH, and accelerated failure time (AFT) models to find the model that best fits the data. The Akaike Information Criterion (AIC) was used to evaluate the performance of models examined in this investigation. Time to teenage childbearing was the study’s outcome variable, while the analysis considered various independent variables. We analyze data from the 2016 National Demographic Health Survey to assess the influence of different risk factors on teenage pregnancy among Ethiopian women.

**Results:**

Out of the 10,274 teenagers (aged between 13 and 19) who participated in the 2016 survey, 6,430 (62.59%) were parents. The study findings revealed that these teenage parents were influenced by various time-related factors before becoming parents. The log-normal AFT model has the lowest AIC value and hence it is the best fit for this data. Results from this model indicated that significant factors influencing the time to teenage childbearing include the age of the household head, current age of the respondents, region, religion, educational attainment, wealth status, intention to use contraception, and recent sexual activity.

**Conclusion:**

This study reveals that 62.59% of surveyed teenagers aged 13 to 19 were parents. Various factors at both the individual and community levels: including the age of the household head, regional differences, religious affiliation, educational level, economic status, contraceptive intentions, and recent sexual activity, determine the time to teenage childbearing. Targeted interventions addressing these factors are essential for reducing teenage pregnancies and supporting adolescent parents effectively.

## Background

Teenage childbearing refers to the phenomenon where adolescents become parents at a young age, typically before the age of 20. It has historically been seen as a social issue because of the possible effects it may have on young moms and their children [1]. According to the United Nations Children’s Fund, teenage pregnancy is defined as a pregnancy in girls between the ages of 13 and 19 [2]. Giving birth to their first child is the primary purpose of reproduction, and it represents a critical turning point in preserving their genetic heritage and advancing the human species [3]. The ages at which women marry and give birth to their first child vary depending on and as a result of demographic parameters [4]. Every year, around 16 million teenage girls between the ages of 15 and 19 give birth. 95% of births worldwide are the result of impoverished nations, and one in ten are the result of young mothers [5]. Particularly in the youngest age groups, adolescent fertility poses significant health risks to the mother and child [6, 7]. The well-being of the mother and her child is impacted by the serious negative health and social effects of early pregnancy and childbearing [8].

Teenage pregnancy carries significant health and social burdens, with unique medical and psychological challenges for both the young mother and society as a whole [2]. Worldwide, 2 million teenagers under the age of 15 and roughly 16 million teenage girls between the ages of 15 and 19 give birth each year [3]. Teenage pregnancy and childbearing raise maternal mortality and morbidity risks, particularly in very young adolescents [3, 9]. Adolescent pregnancy and childbearing have distinct and significant negative effects on society, the world, and individuals [7, 10]. It could also expose adolescent girls to gender-based violence, exclusions, and inequities. Women who have their first child in their teenage years experience faster population growth globally because early pregnancy prolongs the reproductive cycle and boosts fertility. The substantial correlation between adolescent childbearing and low levels of educational success has an adverse effect on women’s status and potential contributions to society on a societal level. Individually, adolescent childbearing is linked to poor outcomes for mother and child health, such as obstructed labor, low birth weight, fetal growth retardation, and a high rate of infant and maternal mortality [7, 10]. Adolescent moms are more likely to have early births, low birth weight babies, and other unfavorable birth outcomes in their offspring [11]. Children born to adolescent mothers are more susceptible to premature birth, low birth weight, and other adverse birth outcomes [11]. The prevalence of adolescent motherhood is much higher in low-income countries when compared to high-income countries [2]. Generally, different literature discusses the global phenomenon of adolescent pregnancy and childbearing, highlighting its extensive repercussions on individuals, society, and the world at large. Millions of teenagers give birth annually, facing significant health risks, including complications during labor and high rates of infant and maternal mortality. Adolescent pregnancy impedes educational attainment and societal contributions, contributing to accelerated population growth. This issue perpetuates a cycle of poor health outcomes across generations, particularly in low-income countries. Addressing adolescent pregnancy necessitates comprehensive strategies involving education, healthcare, and reproductive services to empower adolescents and mitigate its far-reaching consequences globally.

In Africa, nearly one-third of teenagers become pregnant [12]. Ethiopia has one of the highest rates of teenage pregnancy in Africa [13]. The 2016 Ethiopian Demographic and Health Survey (EDHS) found a national prevalence of 16%, with a higher rate in rural areas (15%) compared to urban areas (5%). Notably, many young women in rural Ethiopia become pregnant outside of marriage each year [7].

The region in which an adolescent is located, how rich or poor an adolescent is, literacy, and education are all possible predictors of when an adolescent will start having children at the national level, according to scientific findings. We nevertheless discovered that having ever been married or having ever lived with a man as if married is a consistent predictor of the beginning of childbearing in various locations. Despite the high percentage of teenage childbearing in Ethiopia, factors associated with time to teenage childbearing are not well investigated. Studies are limited at a national level that identify the determinant factor for this higher burden of adolescent childbearing in Ethiopia. This study, therefore, is intended to investigate factors associated with time to teenage childbirth using 2016 EDHS data at the national level.

While research on teenage childbearing exists, it has limitations. Past studies often lack detailed analysis of factors influencing the exact timing of childbearing between ages 13 and 19. Additionally, much of the research is limited in scope, focusing on the presence or absence of the issue and its associated factors rather than the likelihood of pregnancy over time. To address this gap, this study aims to assess both the prevalence of teenage childbearing in Ethiopia and the factors influencing the timing of pregnancy within this age range, we conducted a survival analysis. Survival analysis is a statistical technique that is particularly useful when studying the time it takes for an event to occur. In our case, the “event” is giving birth. By employing survival analysis, we can examine how various factors influence the age at which adolescents in Ethiopia are likely to give birth between ages 13 and 19. Since survival analysis overcomes the drawbacks of traditional regressions like logistic and linear regressions, it was used in this article.

## Methods

### Study area

Ethiopia, situated in the Horn of Africa, is the second most populous nation in Africa after Nigeria. This research was conducted in Ethiopia, which comprises two city governments (Addis Ababa and Dire Dawa) and thirteen regional states (Tigray, Afar, Amhara, Oromiya, Somali, Benishangul-Gumuz, Southern Nations Nationalities and People (SNNP), Gambela, Sidama Region, South West Ethiopia, South Ethiopia Regional State, Central Ethiopia Regional State, and Harari) in Ethiopia [18].

### Study Design

In this research, a community-based cross-sectional study design was employed. This approach involves collecting data from a sample of the population at a single point in time to investigate relationships between variables of interest [19].

### Sampling technique

A stratified two-stage cluster design was utilized to select the 2016 EDHS sample, with the first stage’s sampling units being enumeration areas (EAs). Households were included in the sampling process’ second phase. The interviews were open to all females between the ages of 15 and 49. 16,583 suitable women were given individual interviews from the houses surveyed, and 10,274 received full interviews [7]. 8,839 married women from nine districts and two municipal administrations were included in the current study. Sampling weights have been applied to each case in tabulations to adjust for differences in the probability of selection and interview between cases in a sample, due to either design or happenstance. In DHS surveys, the sample is selected with unequal probability to expand the number of cases available (and hence reduce sample variability) for certain areas or subgroups for which statistics are needed. In this case, weights need to be applied when tabulations are made of statistics to produce the proper representation. When weights are calculated because of sample design, corrections for differential response rates are also made.

### Study Population

Data from the 2016 Ethiopian Demographic and Health Surveys (EDHS) on women who had or had not had childbearing at the ages of 13 and 19 were used in the current study.

**Variables included in the study**: The study on teenage childbearing among women in Ethiopia includes two primary types of variables: the outcome variable and the explanatory variables. The outcome variable represents the survival time for teenage childbearing between the ages of 13 and 19 years. As defined by the United Nations Children’s Fund, an adolescent or teenager is an individual between 13 and 19 years old [2]. In this study, the age at first birth is considered a time variable, measuring the duration from birth to the age of the first childbirth in years. Women who give birth between the ages of 13 and 19 are classified as events. Women who do not give birth between the ages of 13 and 19, or if they give birth to their first child at an age greater than 19, are considered censored. Additionally, based on different literature the study considers several explanatory variables, including demographic characteristics such as current age, marital status, region, place of residence, religion, education status, current employment status, and wealth status. Household head-related factors, such as the sex and age of the household head, are also examined. Furthermore, sexual behavior factors like recent sexual activity and age at the first sexual encounter, as well as reproductive health history variables such as knowledge of contraceptive methods, contraceptive use and intention, and history of terminated pregnancies, are included in the analysis.

**Statistical Analysis**: A data extraction technique was used to extract variables from the EDHS 2016 dataset for children and individual women. The study population was characterized using descriptive measures like graphs and frequency tables after editing and coding. To demonstrate the factors that affect how long it takes adolescents in various countries to start having children, we conducted a survival analysis. The following Survival modes were employed for this research article: non-parametric, semi-parametric PH, parametric PH, and parametric AFT models. The Kaplan-Meier (K-M) and the log-rank test were calculated to show the time of the first birth and to compare the survival time between covariates, respectively. For assessing the PH assumption, we calculated the correlation between the ranking of individual failure times and the Schoenfeld residuals and log-log survival plot. If the PH assumption is met, then the correlation should be near zero [14]. We first analyze our data using Cox proportional hazards, parametric PH models, accelerated failure times, and frailty models. Then, using AIC, the best model was selected.

The choice of survival models in this research article was based on their appropriateness for the study. The non-parametric model was selected for its flexibility in analyzing survival data without making specific assumptions about the underlying distribution. The semi-parametric proportional hazards (PH) model was chosen to allow for the estimation of hazard ratios while relaxing the assumption of a specific functional form for the baseline hazard. The parametric PH model was employed to facilitate the estimation of hazard functions with specific parametric forms, which can provide insights into the underlying survival distribution. Additionally, the parametric accelerated failure time (AFT) model was utilized to explore the effect of covariates on the time-to-event outcome without assuming proportional hazards. These statistical tools were selected to ensure comprehensive analysis and interpretation of the survival data in line with the objectives of the study.

### Survival analysis

The time until an event happens is the outcome variable of interest in the statistical data analysis method known as survival analysis. By the time, we mean the number of years, months, weeks, or days from the start of a person’s follow-up until an event happens. In our case, events refer to teenage childbearing occurring between the ages of 13 and 19, measured by the age at first birth within a year.

### Cox proportional hazards model

The model estimates the hazard ratio, which represents the relative risk of experiencing the event of interest for individuals with different levels of the covariate. The model specification for the Cox proportional hazards model is.

$$h\left(t|x\right)={h}_{0}\left(t\right)*\text{e}\text{x}\text{p}\left({\varvec{\beta }}^{\varvec{{\prime }}}\varvec{x}\right)$$

Where h(t|X) is the hazard rate at time *t* for an individual with covariate values **x**, h_0_(t) is the baseline hazard function, **β** is a vector of regression coefficients, and **x** is a vector of covariate values.

The hazard ratio represents the effect of a one-unit change in the covariate on the hazard rate and is given by: HR = exp(β). Where HR is the hazard ratio, β is the regression coefficient for the covariate, and exp(β) is the exponential of the regression coefficient. A hazard ratio greater than 1 indicates a higher risk of experiencing the event of interest, while a hazard ratio less than 1 indicates a lower risk.

If the proportional hazard assumption criterion of the Cox-PH model is not satisfied, in such an instance, a better approach is to use a parametric model. Accelerated Failure Time (AFT) and parametric PH are other popular parametric models used in survival analysis.

### Parametric proportional hazards model

#### Weibull proportional hazards model

suppose that the values x_1_,…, x_p_ of P explanatory variables, x_1_,…, x_p_ are recorded for each of n individuals. Under the Weibull proportional hazards model, the hazard of death at time t for the *i*^*th*^ individual is.

$${{\mathbf{h}}_{\mathbf{i}}}({\mathbf{t}}) = {\mathbf{exp}}\,({\beta _1}{x_{1i}} + {\beta _2}{x_{2i}} + \ldots {\beta _p}{x_{pi}})\,{h_o}(t)$$

Where h_o_(t) = λαt^(α-1)^, h_o_(t) is the hazard rate at time t, λ is the baseline hazard rate, and α is the shape parameter.

### Gompertz proportional hazards model

Suppose that the values x_1_,…, x_p_ of P explanatory variables, x_1_,…, x_p_ are recorded for each of n individuals. Under the Gompertz proportional hazards model, the hazard of death at time t for the *i*^*th*^ individual is expressed as:

$${{\mathbf{h}}_{\mathbf{i}}}({\mathbf{t}}) = {\mathbf{exp}}\,({\beta _1}{x_{1i}} + {\beta _2}{x_{2i}} + \ldots {\beta _p}{x_{pi}})\,{h_o}(t)$$

Where h_o_(t) = λ*exp(αt), h_o_(t) is the hazard rate at time t, λ is the baseline hazard rate, and α is the shape parameter.

#### Note

The proportional hazards assumption for the above two models implies that the hazard ratio between any two individuals is constant over time and is equal to the exponential of a linear combination of covariates:

### Accelerated failure time (AFT)

When the assumption of Cox PH is violated, an alternative to parametric models and Cox PH for the analysis of survival time data is the accelerated failure time model. Instead of measuring a hazard, we can estimate the direct impact of the covariates on survival time under AFT models. The Weibull, log-logistic, log-normal, and gamma distributions are among the common distributions used in the AFT model.

### Weibull AFT model

The Weibull distribution is unique among distribution families in that it may be parameterized as an AFT model. For time-to-event data, the Weibull distribution is an extremely flexible model. Its hazard rate is either constant, monotonically declining, or increasing [15]. The survival and hazard functions of the Weibull AFT model, respectively, are given by

$${S}_{i}\left(t\right)=\text{exp}\left\{-\text{exp}\left(\frac{\text{log}t-\mu -{\beta }_{1}{x}_{1}-\dots -{\beta }_{p}{x}_{p}}{\sigma }\right)\right\}$$

$${h}_{i}\left(t\right)=\frac{1}{\sigma t}\text{exp}\left(\frac{\text{log}t-\mu -{\beta }_{1}{x}_{1}-\dots -{\beta }_{p}{x}_{p}}{\sigma }\right)$$

Where β1, …, βp are the coefficients, The acceleration factor is exp(β’X), which represents the effect of the covariates on the survival time.

### Log-logistic AFT model

The monotonic nature of the Weibull hazard function is one of its drawbacks. Nonetheless, under certain circumstances, a hazard function may reverse course. In these kinds of cases, the most popular AFT model is the log-logistic distribution. It is a non-monotonic hazard function. The survival and hazard functions of the log-logistic AFT model, respectively, are given by

$${S}_{i}\left(t\right)={\left\{1+\text{exp}\left(\frac{\text{log}t-\mu -{\beta }_{1}{x}_{1}-\dots -{\beta }_{p}{x}_{p}}{\sigma }\right)\right\}}^{-1}$$

$${h}_{i}\left(t\right)={\frac{1}{\sigma t}\left\{1+\text{exp}\left(\frac{\text{log}t-\mu -{\beta }_{1}{x}_{1}-\dots -{\beta }_{p}{x}_{p}}{\sigma }\right)\right\}}^{-1}$$

Where β_1_, …, β_p_ are the coefficients, The acceleration factor is exp(β’X), which represents the effect of the covariates on the survival time.

### Log-normal AFT model

It is possible to utilize the log-normal distribution as a model for survival data because it is also defined for random variables that take positive values. The survival function of the log-normal AFT model is given by

$${S}_{i}\left(t\right)=1-\varphi \left(\frac{\text{log}t-\mu -{\beta }_{1}{x}_{1}-\dots -{\beta }_{p}{x}_{p}}{\sigma }\right)$$

Where β1, …, βp are the coefficients, $\varphi (.)$ is the standard normal distribution function. The acceleration factor is exp(β’X), which represents the effect of the covariates on the survival time.

### Gamma AFT model

The gamma distribution is quite similar to the Weibull, and inferences based on either model will often be very similar. The probability density function of the gamma model.

$$f(t) = \frac{{\alpha {\lambda ^{\alpha \gamma }}}}{{\tau (\gamma )}}t\alpha {\gamma ^{ - 1}}\exp [ - {(\lambda t)^\alpha }]\,\,\,t > o,\gamma > o,\lambda > o,\alpha > o,$$

Where 𝛾 is the shape parameter of the distribution. The exponential, Weibull, and lognormal models are all special cases of the generalized gamma model. The generalized gamma distribution becomes the exponential distribution if 𝛼 = 𝛾 = 1, the Weibull distribution if 𝛾 = 1, and the log-normal distribution if 𝛾 → ∞.

### Model comparison

The selection and comparison of models is one of the most frequent issues in statistical practice. In this work, we used the information criterion: Akaike information criterion (AIC), and − 2 log likelihood (− 2LL) for comparison of the performance of semi-parametric, parametric, and AFT models. Accordingly, the best model was the one with the smallest information criterion indicating the minimum loss of information. Moreover, the estimates of semi-parametric, parametric, and AFT models were compared with non-parametric estimates (as discussed by [16, 17]) to assess model fit.

### Data processing

First, the data was checked for completeness and consistency. Then it was coded and entered into STATA and R software.

## Result

The main characteristics of the women respondents identified in the study area are presented in Table 1. 10,274 women in all were taken into account for the analyses in this study. In this study, the women’s functional status is noted under two headings: not teenage childbearing (censored) and teenage childbearing (event). The descriptive table out of 10,274 women shows that 3,844 (37.41%) of them were not teenage childbearing at the end of the trial, whereas 6,430 (62.59%) of them were teenage childbearing.

As we can see from the result in Table 1, out of the total of 10,274 women respondents included in the study about 73.4% of respondents were from rural areas while 26.6% were from urban areas. From the total sample, rural residents appear to have a higher teenage childbearing rate (49.3%) than urban residents (13.3%).

Regarding religion, 37.3% of the respondents were Orthodox, 18.8% were protestant, 42.7% were Muslim, and 1.29% were other religions. The proportion of teenage childbearing is higher for women whose religion is Muslim (27.8%), followed by respondents from Orthodox (22.3%), while the lowest proportion of teenage childbearing was from protestant (11.7%) and other religions (0.8%).

Regarding education level, 60.3% had no education, 26.6% had primary education, 8.5% had secondary education, and 4.7% had higher education. From this, the teenage childbearing proportion was highest for those women who had no education (40.2%), followed by those who had primary education(17.3%), while the lowest proportion of teenage childbearing was recorded for women who had secondary(3.8%) and higher education(1.8%) respectively.

About the wealth status of respondents, 44.6% were poor, 13.6% were middle, and 41.8% were rich. From this, the teenage childbearing proportion was highest for those women who came from poor wealth status (29.7%), followed by those women who came from rich wealth status (23.9%), while women who came from the middle wealth status had the lowest proportion of teenage childbearing (9.1%).

About 64.89% of respondents had active sexual activity in the last four weeks, 9.28% of respondents had not been active in the last four weeks- postpartum, and 25.83% had not been active in the last four weeks-not postpartum. From the total respondents, the teenage childbearing proportion for respondents who had active sexual activity in the last four weeks was 41.24%, followed by those who had no active sexual activity in the last four weeks not postpartum (25.83%), and the proportion of teenage childbearing respondents who had not active sexual activity in the last four weeks—postpartum is 9.28%.

As shown by the chi-square and independent t-test results in Table 1, the variables: region, place of residence, religion, educational status, sex of HH, wealth status, current marital status, currently/formerly never in the union, age at first sex, contraceptive use, and intention have a significant association with time to teenage childbearing, whereas respondent current work, knowledge of any contraceptive method, and ever having terminated pregnancy have no significant association with time to teenage childbearing. That is, the insignificant categorical variables are not included in the log-rank test analysis.

**Table 1:** Summarization of factors associated with teenage childbearing of respondents

Variables	Categories	N*o* of Teenage childbearing (%)	Total (%)	Chi-square and/or independent t-test
Region	Tigray	717 (6.98%)	1,107 (10.77%)	0.000
	Afar	593 (5.77%)	835 (8.13%)	
	Amhara	800 (7.79%)	1,160 (11.29%)	
	Oromia	926 (9.01%)	1,366 (13.30%)	
	Somali	585 (5.69%)	1,002 (9.75%)	
	Benishangul G.	561 (5.46%)	804 (7.83%)	
	SNNPR	746 (7.26%)	1,225 (11.92%)	
	Gambela	498 (4.85%)	756 (7.36%)	
	Harari	341 (3.32%)	605 (5.89%)	
	Addis ababa	281 (2.74%)	760 (7.40%)	
	Dire daw	382 (3.72%)	654 (6.37%)	
Place of residence	Urban	1,366 (13.30%)	2,732 (26.59%)	0.000
	Rural	5,064 (49.29%)	7,542 (73.41%)	
Religion	Orthodox	2,291 (22.30%)	3,829 (37.27%)	0.000
	Protestant	1,205 (11.73%)	1,929 (18.78%)	
	Muslim	2,852 (27.76%)	4,383 (42.66%)	
	Others	82(0.8%)	133 (1.29%)	
Education status	No education	4,132 (40.22%)	6,190 (60.25%)	0.000
	Primary	1,775 (17.28%)	2,731 (26.58%)	
	Secondary	392 (3.82%)	873 (8.50%)	
	Higher	131 (1.28%)	480 (4.67%)	
Current age	Mean	31.2902	31.95678	0.000
Sex of household head	Male	4,752 (46.25%)	7,438 (72.40%)	0.000
	Female	1,678 (16.33%)	2,836 (27.60%)	
Age of household head	Mean	39.62986	40.35468	0.000
Wealth status	Poor	3,049 (29.68%)	4,577 (44.55%)	0.000
	Middle	931 (9.06%)	1,400 (13.63%)	
	Rich	2,450 (23.85%)	4,297 (41.82%)	
Current marital status	Never in union	60 (0.58%)	120 (1.17%)	0.001
	Married	5,567 (54.19%)	8,839 (86.03%)	
	Widowed	292 (2.84%)	443 (4.31%)	
	Divorced	511 (4.97%)	872 (8.49%)	
Currently/formerly/never in union	Never in union	60 (0.58%)	120 (1.17%)	0.007
	Currently in union/living with a man	5,567 (54.19%)	8,839 (86.03%)	
	Formerly in a union/living with a man	803 (7.82%)	1,315 (12.80%)	
Respondent’s currently working	No	3,324 (32.35%)	5,255 (51.15%)	0.152
	Yes	3,106 (30.23%)	5,019 (48.85%)	
Recent sexual activity	Active in last 4 weeks	4,237 (41.24%)	6,667 (64.89%)	0.000
	Not active in last 4 weeks – postpartum	544 (5.29%)	953 (9.28%)	
	Not active in last 4 weeks - not postpartum	1,649 (16.05%)	2,654 (25.83%)	
Knowledge of any method	No	268 (2.61%)	419 (4.08%)	0.552
	Yes	6,162 (59.98%)	9,855 (95.92%)	
Contraceptive use and intention	Does not intend to use	3,096 (30.13%)	4,824 (46.95%)	0.004
	Non-user - intends to use later	1,769 (17.22%)	2,920 (28.42%)	
	Using contraceptive method	1,565 (15.23%)	2,530 (24.63%)	
Ever had a terminated pregnancy	No	5,742 (55.89)	9,172 (89.27)	0.911
	Yes	688 (62.59)	1,102 (10.73)	
Age at first sex	**Mean**	15.25739	16.65233	0.000

### Log-rank test

The survival experience of teenage women among various parameters using Kaplan-Meier estimates is presented in Table 2. The comparison of the survival function among different groups of covariates was examined using the log-rank test (Table 2). Based on the results, we noted that women living in Gambella, those rural residents, those with lower education, poor wealth status, recently active sexual activity, divorce marital status, and don’t intend to use contraceptive methods were significantly associated with smaller survival time of the first birth of teenagers childbearing.

**Table 2:** Comparison of survival experience on timing first birth of teenager’s childbearing on Socio-demographic characteristics, sexual and reproductive health history of the respondent

Variables	Categories	Mean childbearing age	Log-rank t-test	Log-rank *P*-value
Region	Tigray	25.02	306.20	0.000
	Afar	23.1		
	Amhara	22.93		
	Oromia	23.49		
	Somali	24.81		
	Benishangul G.	21.15		
	Snnpr	23.94		
	Gambela	22.48		
	Harari	25.89		
	Addis Ababa	31.47		
	Dire Daw	24.31		
Place of residence	Urban	28.29	207.33	0.000
	Rural	23.69		
Religion	Orthodox	26.05	29.44	0.000
	Catholic	21.1		
	Protestant	25.11		
	Muslim	24.43		
	Others	22.74		
Education status	No Education	24.39	354.95	0.000
	Primary	24.19		
	Secondary	29.06		
	Higher	33.68		
Sex of household head	Male	24.4	11.23	0.000
	Female	26.13		
Wealth status	Poor	23.82	85.05	0.000
	Middle	23.77		
	Rich	26.68		
Current marital status	Never in Union	28.41	15.00	0.002
	Married	25.33		
	Widowed	24.11		
	Divorced	24.56		
currently/formerly/never in union	Never in Union	28.41	7.54	0.023
	Currently in Union/Living with A Man	25.33		
	Formerly In Union/Living With A Man	25.23		
Recent sexual activity	Active In Last 4 Weeks	25.17	16.78	0.000
	Not Active in Last 4 Weeks – Postpartum	26.32		
	Not Active in Last 4 Weeks - Not Postpartum	25.28		
Contraceptive use and intention	Does Not Intend to Use	24.94	23.98	0.000
	Non-User - Intends to Use Later	25.16		
	Using Contraceptive Method	25.27		

### Cox PH analysis

To determine the sexual and reproductive health history and demography factors that are associated with the time to teenage childbearing of women, we first fitted a univariate Cox proportional hazard model for each potential risk factor before proceeding to more complicated models. Variables having a p-value less than or equal to 0.25 in the univariate analysis were considered in the multivariable model. Then, the full multivariable Cox proportional hazard model was fitted, including all the potential covariates that were significant at 25% at the univariate level. For multivariable analysis, the forward selection method was used, and variables with a P-value less than or equal to 5% were selected as significant covariates. The final multivariable Cox PH model identified the following significant variables: current age of respondents, age at first sex, region, religion, educational status, wealth status, contraceptive use and intention, and recent sexual activity.

### Checking the linearity of continuous variables and influential observation in the model

**Fig. 1:**
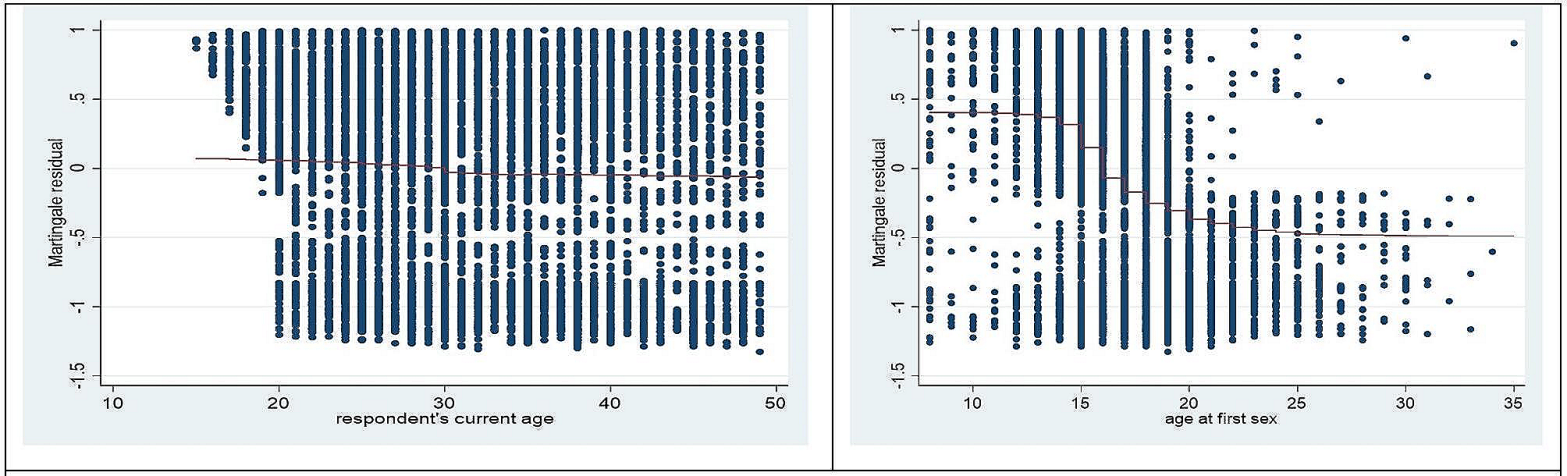
The plot of martingale residual against the Covariates

**Fig. 2:**
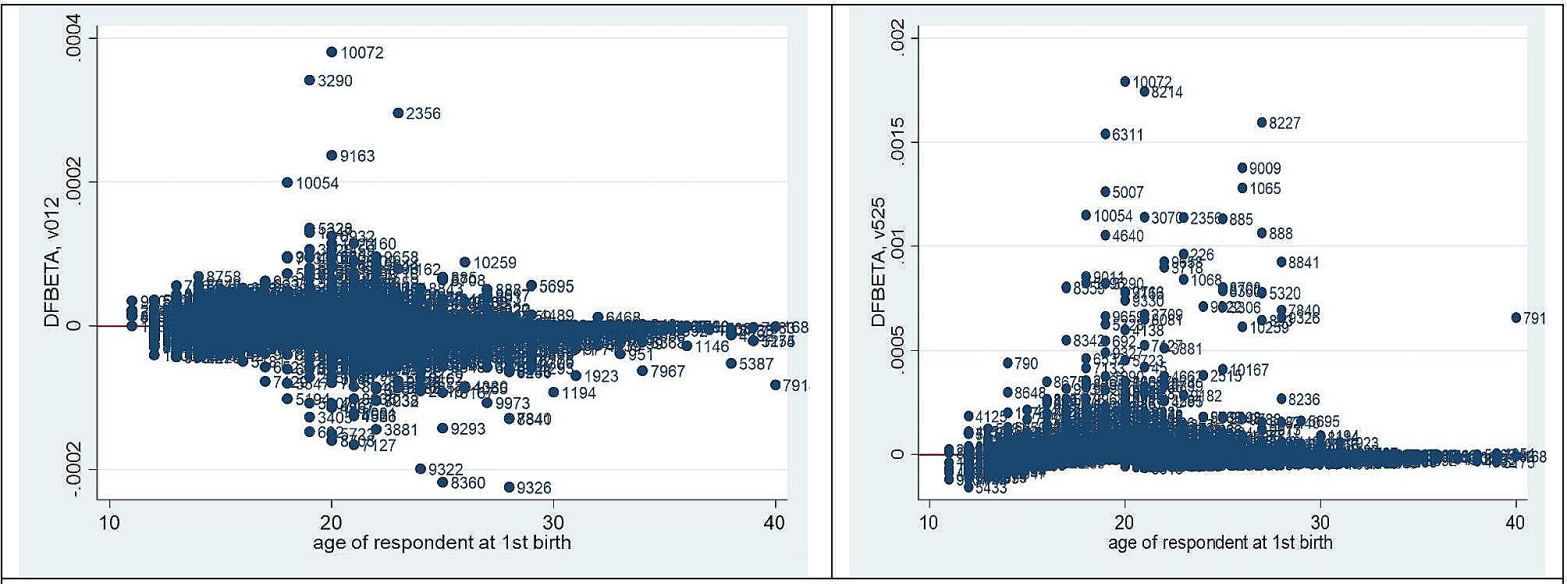
The plot of DFBETA of the Covariates

Plotting the martingale residuals against a covariate can show whether the function form is accurate. These are signs of roughly linear covariate behavior. Thus, a plot of the martingale residuals versus the linear predictor is used to detect outliers. Figure 1 depicts the output for the martingale residual plotted against the respondents’ covariates of current age and age at first sex. These are indicators of approximately linearity in the covariates and there is no influential observation in the data. As we have seen from Fig. 2 DFBETA plot, there is no influential observation in the data set.

### Model adequacy checking of Cox PH

**Fig. 3:**
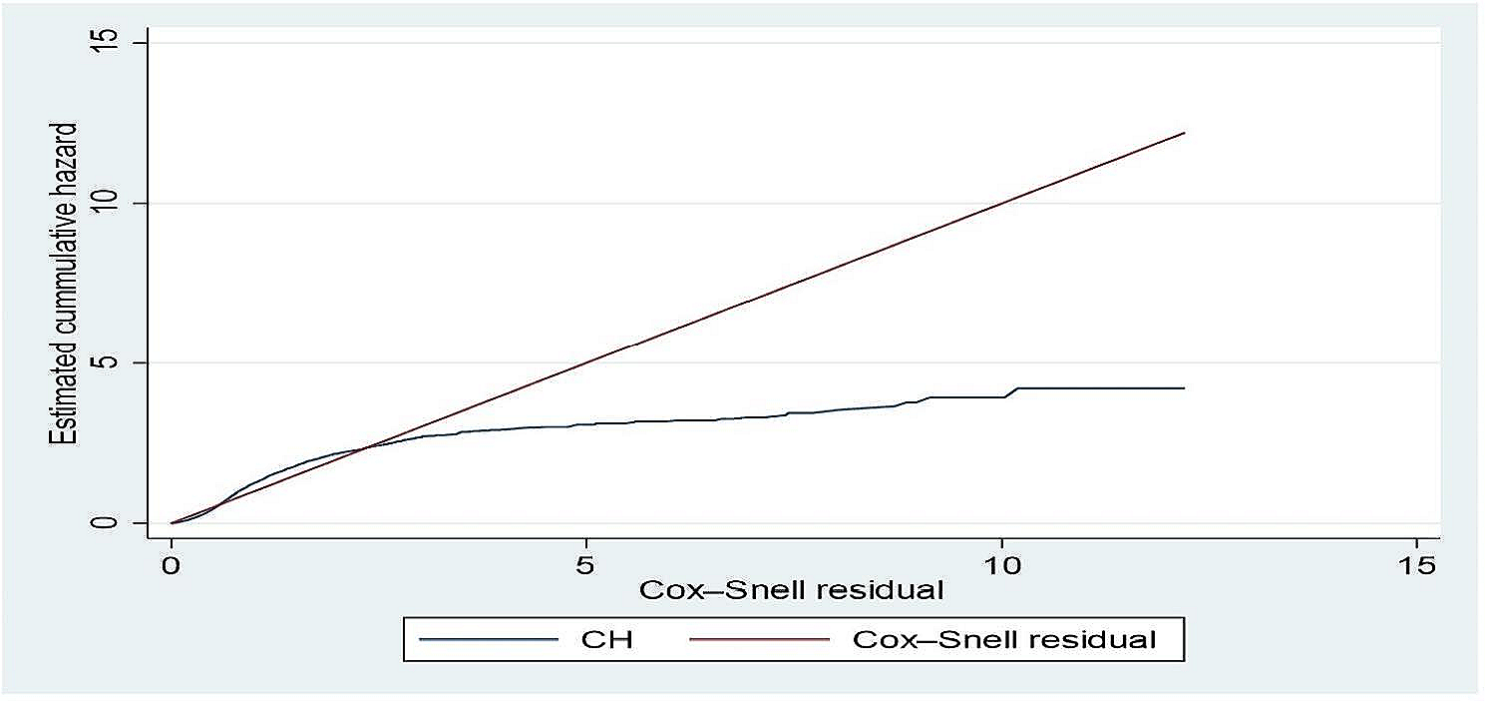
Cox-Snell residual plot

We assessed the goodness-of-fit of the Cox PH model by using the Cox-Snell residual plot. The plot of the estimated cumulative hazard function of the Cox-Snell residuals against the Cox-Snell residuals is presented in Fig. 3 below. In Fig. 3, the blue line is the estimation of Cox-Snell residuals while the red line is the origin with a slope equal to 1. The plot suggests that the Cox PH model does not fit the straight line adequately.

### Testing PH Assumption

**Fig. 4:**
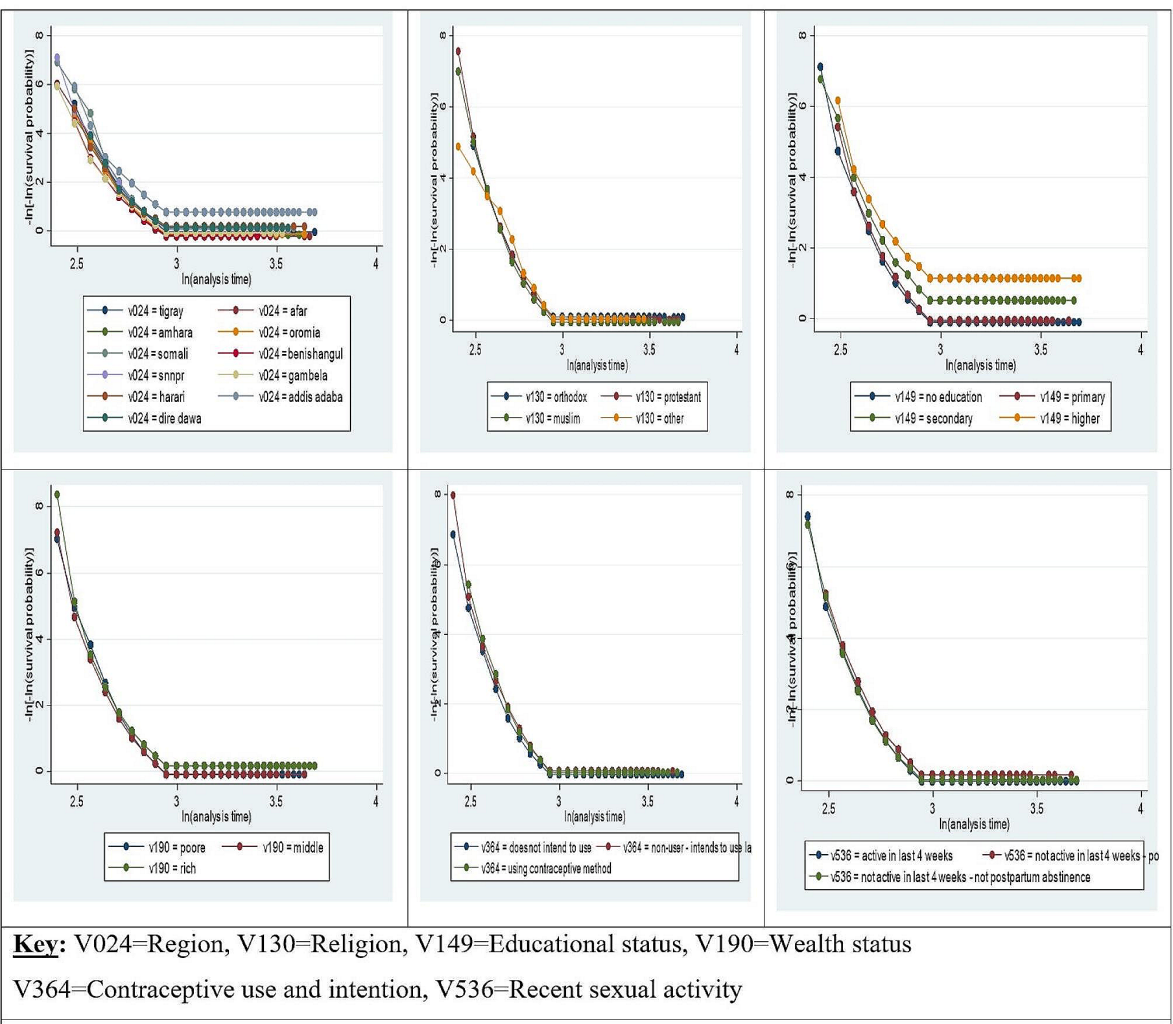
Test of proportionality assumption for categorical variables

The final model is based on the major assumption that the hazards between groups are proportional. To test the assumption of proportionality, the scaled Schoenfeld residuals and log-log survival plots have been used. The results in Table 3; Fig. 4 show that the p-values corresponding to the covariates region, place of residence, wealth status, contraceptive use, and intention are less than 0.05, indicating that the p-value of the rho statistic is less than 5%. A given covariate indicates the rejection of the null hypothesis of the proportionality of the Cox proportional hazard model; the assumption of proportional hazard is not satisfied for those variables. Conversely, the p-values are greater than 0.05 for all the remaining covariates, like religion, educational status, and recent sexual activity. One way of accommodating non-proportional hazards in a model is to use the parametric PH and AFT models. Hence, the parametric PH and AFT models are more appropriate choices.

**Table 3:** Test PH assumption by using Scaled Schoenfeld residuals

Covariates	Chi-square value	Df	*p*-value
Region	5.08e-01	1	0.0022
Religion	5.08e-01	1	0.4762
Educational status	5.77e-04	1	0.9808
Wealth status	9.86e + 00	1	0.0017
Contraceptive use and intention	2.31e + 01	1	1.6e-06
Recent sexual activity	1.73e + 00	1	0.1887
GLOBAL	4.37e + 01	6	8.6e-08

In survival analysis, Akaike’s information criterion (AIC) or the − 2 log-likelihood function (-2 logL) can be used to compare a variety of models. The values of AIC and − 2 logL for the semi-parametric survival (cox PH) model and all parametric survival models are given in Table 4. The result of this study shows that the lognormal parametric AFT model provides a lower AIC value among other models and hence the lognormal parametric AFT model gives more suitable results for survival data in the presence of the non-proportional hazards assumption.

The study shows that when faced with non-proportional hazard assumptions, the lognormal parametric model, as indicated by the Akaike Information Criterion (AIC), is more appropriate for this survival data. This preference is explained by its ability to capture a variety of survival patterns, including those with changing rates of hazards over time, which are typical of situations involving non-proportional hazards. The superiority of the lognormal model is further supported by its stronger model fit, robustness in estimation, and adherence to statistical criteria like AIC. In the end, the lognormal model provides a parsimonious but efficient representation of survival dynamics in the presence of non-proportional hazards and enables more accurate statistical inference (See Table 4).

**Table 4:** Information Criterion Statistics for Semi-parametric, Parametric, and AFT Models

Models	Number of parameters	Log-likelihood	AIC
Cox PH	24	-55202.697	110453.4
Exponential PH	25	-10041.475	20132.95
Weibull PH	26	-4676.1204	9404.241
Gompertz PH	26	-5883.4512	11818.9
Exponential AFT	25	-10041.475	20132.95
Weibull AFT	26	-4676.1204	9404.241
Lognormal AFT	**26**	**-3632.7675**	**7317.535**
Loglogistic AFT	26	-3706.3514	7464.703
Gamma	27	-11419.009	22892.02

Note: PH = Proportional Hazard, AFT = Accelerated Failure Time

### Factors influencing time to teenage childbearing

Analysis results for modeling time to teenage childbearing using the lognormal AFT model are presented in Table 5. Based on the results, we observed that the variables current age, age of household, region, education level, wealth status, contraceptive use, intention, and recent sexual activity are significant factors influencing the time to teenage childbearing. The adjusted time ratio (aTR) for the current age of the respondent and the age of the household head is 1.0052 and 1.0008, respectively. An increase in the current age of the respondent and the age of the household, an increase in survival time (i.e. age at first birth) by factors of 1.0052 and 1.0008, respectively. The result further showed that the age at first birth for women from the Gambela region (aTR = 0.9597) was significantly smaller than for those women from the Tigray region, while the age of first birth for women from Somalia (aTR = 1.1065), Harari (aTR = 1.0587), Addis Ababa (aTR = 1.1674), and Dire Dawa (aTR = 1.0539) regions was significantly higher than for those women from Tigray.

With regard to educational status, the mean age at first birth for women with primary (aTR = 1.0241), secondary (aTR = 1.1533), and higher (aTR = 1.3184) educational status was significantly lower than for women with no education. Concerning wealth status, the mean age at first birth for affluent women (aTR = 0.9778) is significantly lower as compared to poor women. According to recent sexual activity of women, being not active in the last 4 weeks postpartum increases the median time to childbearing by 1.0615, while being not active in the last 4 weeks, irrespective of postpartum status, accelerates the time to teenage childbearing by 0.9869.

**Table 5:** Results of lognormal parametric AFT model

Variables	aTR	95% CI for aTR	*P*-value
**Current age**	1.0052	1.004–1.006*	0.000
**Age of household head**	1.0008	1.0003-1.00*	0.001
**Region (Tigray reff.)**			
Afar	1.0194	0.99–1.05	0.196
Amhara	0.9796	0.96-1.00	0.077
Oromia	1.0030	0.979–1.03	0.808
Somali	1.1065	1.07–1.14*	0.000
Benishangul	0.9775	0.95–1.003	0.095
SNNP	1.0272	1.0-1.06	0.050
Gambela	0.9597	0.93–0.99 *	0.007
Harari	1.0587	1.03–1.09*	0.000
Addis Adaba	1.1674	1.13–1.20 *	0.000
Dire Dawa	1.0540	1.02–1.08]*	0.000
**Religion (Orthodox reff.)**			
Protestant	1.0048	0.98–1.02	0.644
Muslim	0.9884	0.97-1.00	0.173
Other	1.0521	1.00-1.10 *	0.045
**Educational status (No educ. reff.)**			
Primary	1.0241	1.01–1.04*	0.001
Secondary	1.1533	1.13–1.17*	0.000
Higher	1.3183	1.27–1.36*	0.000
**Wealth status (poor reff.)**			
Middle	0.9887	0.97–1.01	0.192
Rich	0.9778	0.96–0.99*	0.002
**Contraceptive use and intention (does not intend to use reff.)**			
Non-user - intends to use later	1.0429	1.03–1.059*	0.000
Using contraceptive method	1.0537	1.038–1.069*	0.000
**Recent sexual activity (active in last 4 weeks reff.)**			
Not active in last 4 weeks – postpartum	1.0615	1.04–1.08*	0.000
Not active in last 4 weeks - not postpartum	0.9869	0.97- 0.99*	0.049

Keys: *p < 0.05 in aTR; aTR = Adjusted time ratio

## Discussion

Based on our findings, intervention designs aimed at significantly reducing early childbearing among young people should consider context-specific elements. The combination of factors explaining the onset of childbearing varies across different regions. Since survival analysis addresses the limitations of traditional regression methods such as logistic and linear regressions, it was employed in this study. Survival analysis allows for the evaluation of the relationship between event survival times while considering censored observations. This study utilized survival analysis to examine the time until adolescent childbearing and its contributing factors. According to our findings, various factors influence the duration it takes for teenagers to become parents, including their current age, household age, geographic area, educational level, financial situation, intention to use contraception, and recent sexual activity.

There were variations in the age at which first births occurred across regions of Ethiopia. This may be due to differences in culture, societal norms, and values. The literature consistently agrees that there are geographical variations in the timing of first births [11, 18, 19]. According to this study, women in the Afar, Amhara, Oromia, Benshangule, and Gambla regions are more likely to give birth early than those in the Tigray region. On the contrary, women living in Harari, Somalia, SNNP, Addis Ababa, and Dire Dawa regions have a lower likelihood of early childbirth than women in the Tigray Region. This is consistence with the findings of a Vietnam study that found women from the North to have significantly higher ages at first birth than women from the South [19].

We also found that women who had ever used contraceptives had a delayed first birth and thus had a lower likelihood of early first birth than those who never used contraception. This could be explained by the fact that those who used something to prevent pregnancy were in control of their fertility and chose to postpone childbearing. This was in agreement with previous studies [20, 21].

Education was another important factor utilized that statistical analysis to determine when adolescent childbearing occurred. A higher level of education postpones having children as people focus on their careers and personal development. According to the study’s findings, women with primary, secondary, and higher education were less likely to become pregnant in their teens than those who didn’t have any education. Studies conducted in various parts of Africa [22], Pakistan [9], and Nepal [18] all support this conclusion. This may be because more educated girls, through school, the internet, media, and literature, have a better grasp of and attitudes toward possible health and lifestyle issues than less educated girls [23].

The wealth status of the household is another major factor in determining the time to teenaged childbearing. Participants in the rich category are less likely to have a positive tendency to a predisposition towards early pregnancy and motherhood than the poor. This is similar to studies done in Nigeria [10] and Ethiopia [1]. The wealth status of the individual, which may indicate access to resources and support, can influence the timing of childbearing. Studies done in Africa and the United States of America have shown that low-income girls may consider early marriage and sex as a means of income generation to lead their daily lives with money scarcity [10].

The current age of teenagers and the age of household heads were also negatively associated with time to teenage childbearing. The younger the individual is, the higher the likelihood of teenage childbearing. This is supported by previous studies [10]. Similarly, Females who came from younger household heads had more likelihood of early childbearing than the older household heads. This may indicate the level of support and resources available to the individual. If the household is relatively young, it may suggest that the individual is more likely to have children at a younger age.

## Conclusion

In Ethiopia, more than 60% of young girls aged 15 to 19 have already begun having children. Despite a decline in recent years, a substantial proportion of teenagers had children. Oromia region had the greatest proportion of adolescent childbearing followed by the Amhara region while Addis Ababa city had the lowest rate. The majority of women in rural regions who do not use or intend to use contraception have the chance of childbearing at teen ages. Teenagers who start sex before the age of 18 are more likely to have a child than those who are in late adolescence, married, or live with a partner. The study employs different Cox, parametric PH, and parametric AFT models including Exponential PH, Weibull PH, Geopertze PH, Exponential AFT, Lognormal AFT, Loglogistic AFT, and Gamma models. Among these candidate models, the Lognormal AFT model provides the lowest AIC value. This indicates that the Lognormal AFT model is the best model. In conclusion, this study found that the current age of respondents, age of household head, region, religion, educational level, wealth status, contraceptive use and intention, and recent sexual activity are the significant factors of time to teenage childbearing. In order to lower teenage childbearing and associated difficulties, intervention programs targeted at enhancing adolescent understanding and use of contraceptives as well as avoidance of early sexual beginning are crucial. The government should also ensure easy access to contraception for teenagers and conduct community outreach programs to raise awareness about the consequences of teenage pregnancy and the importance of delaying parenthood. The government can engage with various religious and ethnic communities in a culturally sensitive manner. Furthermore, the government should address socio-economic factors that contribute to early childbearing by providing economic opportunities for young people and their families. This could include job training programs and microfinance initiatives aimed at alleviating poverty.

## Data Availability

The datasets used and/or analyzed during the current study are available from the corresponding author upon reasonable request.
